# Change in depressive symptoms over higher education and professional establishment - a longitudinal investigation in a national cohort of Swedish nursing students

**DOI:** 10.1186/1471-2458-10-343

**Published:** 2010-06-15

**Authors:** Anna Christensson, Bo Runeson, Paul W Dickman, Marjan Vaez

**Affiliations:** 1 Department of Clinical Neuroscience, Karolinska Institutet, Stockholm, Sweden; 2 Department of Medical Epidemiology and Biostatistics, Karolinska Institutet, Stockholm, Sweden; 3 Unit for Clinical Cancer Epidemiology, Department of Oncology and Pathology, Karolinska Institutet, Stockholm, Sweden

## Abstract

**Background:**

There are indications of a high prevalence of psychological distress among students in higher education and also that distress increases over the course of study. However, not all studies on student distress controlled for sociodemographic differences and few followed development of distress over an extended period through professional establishment. We investigated if there is an independent effect of time in education and the first two years in the profession on depressive symptoms and mapped change over the period in a national cohort of students.

**Methods:**

Data came from LANE, a nation-wide longitudinal panel survey of Swedish nursing students (N = 1700) who responded to annual questionnaires over five years from 2002 to 2007. Depressive symptoms were measured by the Major Depression Inventory and change over time analysed in a linear mixed effects model for repeated measures.

**Results:**

There was a significant change in level of depressive symptoms over time: an increase from the first to later years in education and a decrease to levels similar to baseline after graduation and a year in the profession. The change in symptoms remained significant after adjustment for sociodemographic factors (p < 0.01). Symptom levels differed due to age, gender, household composition and prior nurse assistant training but change over time was similar in all groups. The correlation among the repeated measures, representing within individual correlation over time, varied between 0.44-0.60.

**Conclusions:**

The findings indicate an independent but transitional effect of time in education and professional establishment on depressive symptoms. We think heightened distress over education abates as the graduate accommodates to the profession. Nevertheless, within education, the differences in depressive symptoms associated to demographic factors can help identify student groups more vulnerable to distress. Also, as individual differences in distress seem to persist over time, perhaps students highly distressed in the beginning of education can be helped by awareness among educators of the elevated levels of distress in late education.

## Background

Research on mental health in university students has shown that they are subject to high levels of psychological distress and depression, with reports of 10-50% clinically afflicted [1-5]. In addition, longitudinal studies and studies that stratified for stage in education indicate an increase of psychological distress and depression over the course of higher education [6-9]. These findings are a cause for concern given the number of individuals affected (approximately 25% of the population between ages 20-29 within the OECD-countries [10]).

However, many studies were performed in samples that did not allow control for demographic factors and different methods were applied to measure psychological distress or depression. When self-report instruments validated to measure a DSM-IV based definition of depression in random samples of students large enough to take age and gender differences into account, results of depression prevalence were similar to those from a general population [11-13]. This brings to question whether students are subject to distress specific to education, or whether it is better explained by the sociodemographic make up of the group; as emerging adults in transition from adolescence to adulthood [14]. In contrast, the findings of increased psychological distress over education seem to indicate an effect of time in education on distress. A change in distress over education also raises the question of development through an extended period after graduation, during professional establishment.

In nursing students, results from studies that stratified for stage in education appear similar to other student samples in that they show an increase of distress and/or decrease in adaptive coping skills by year in education [15-17]. In newly qualified nurses the transition from education to professional work has been shown stressful [18-21]. Few studies investigated depression or depressive symptoms, but exceptions are Ahmadi, Haack, Ross and Williams [22-25].

We used data from LANE, a longitudinal study of a nation-wide sample of Swedish nursing students, to investigate 1) if there is an effect of time in higher education and establishment in the profession on depressive symptoms, independent of sociodemographic factors and 2) to map the pattern of change in depressive symptoms over the period. The longitudinal design with follow up of the same individuals allowed us to control effects on the individual level on overall change and also to investigate change in individuals compared with overall change. The extended follow-up over the transition from education to professional establishment gave us an uncommon opportunity to connect mental health development during education with mental health in the occupation.

## Methods

### Study population

**Figure 1:**
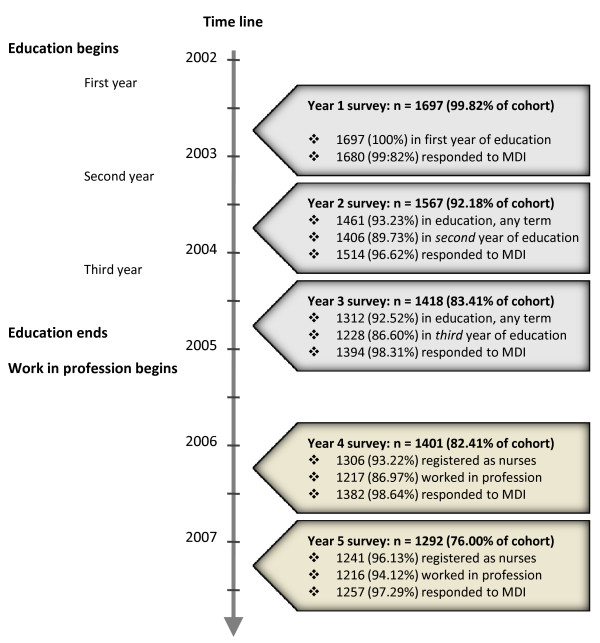
Description of the cohort over time*. *Response rates compared with the defined cohort of 1700; n and (%) responders in education/graduated and registered as nurses; n and (%) with information on depressive symptoms at each time point.

LANE is conducted by a research group at Karolinska Institutet to monitor health development among nursing students and newly qualified nurses [26]. In autumn 2002, all students registered in their second term at any of 26 universities and colleges offering nursing education in Sweden (N = 2331) were asked to participate. Of these 1700 (73%) responded and make up a cohort followed through annual questionnaires. The present analysis includes five data collections and the majority of respondents were expected to have two years professional experience at last follow-up (see Figure 1.). LANE was approved by the Ethical Review Board at Karolinska Institutet.

At baseline there was no difference in mean age between participants and non-responders (28.42 versus 28.36 years), but women were more likely than men to partake (74.1% versus 64.3%, p = < 0.01). The participation rate ranged from 46.3% to 85.9% between universities. Due to technical mistakes three students were excluded from the baseline dataset, yielding a response rate of 1697 year one. These respondents were included from year two (total N of cohort = 1700).

### Measures

Information on depressive symptoms was gathered each year and measured by the Major Depression Inventory (MDI) [27,28] containing 12 items that follow both DSM-IV and ICD-10 symptoms of depression. The instrument can be analysed as a summated scale after collapsing the 12 items into 10 and, using an algorithm, as a dichotomous measure of presence versus absence of depression. All items were answered on a 4-point Likert scale indicating frequency of symptoms during the past two weeks with the answer format almost never/never, a minor part of the time, most of the time, all the time. The items were coded from 0 (almost never/never) to 3 (all the time) yielding a summated scale with a possible range of 0-30. Cronbach's alpha for the MDI varied between 0.88 and 0.89 over the five time points, which is similar to prior validation studies [27]. At baseline the mean level of depressive symptoms according to the diagnostic algorithm was 17.7 (SD 4.1) among those with depression, and 6.6 (SD 3.9) among non-depressed, compared with an overall mean of 7.73 (SD 5.29).

### Included factors

To test the hypothesis of an independent effect of time in higher education and professional establishment on depressive symptoms we adjusted for confounding by age, gender, prior higher education, former training as assistant nurse at high school level, work experience, university (collected at baseline), household composition (collected all years), and stratified for completion of the nursing degree (collected years four and five). We chose to not to adjust for any self reported evaluations of education and work in the argument they may be indicators of distress themselves.

Baseline age was categorised as 20-24, 25-29, 30-34, and 35 years and above. For prior higher education respondents were divided into no experience, less than, and more than a year's experience. Prior work experience was defined as at least six months' continuous employment within or outside health care. In analysis we divided between those with no experience, experience from either within or outside health care, and from both within and outside health care. Household composition was classified in five groups: living with parents, single, single with children, cohabitant/married and cohabitant/married with children.

### Statistical analyses

We investigated response profiles over time using a linear mixed effects model for repeated measures. The analyses were run on a) all cohort members, and b) the subset of students who in year four remained in the study and reported that they had graduated (to assess the effect of drop out over time and the missing at random assumption underlying the statistical model). The respondents were clustered within universities and we added a random effect for university to investigate variance induced at this level each point in time. As this was small (0.08 in a multivariable model containing all other factors) compared with variance at the individual level (23.24 - 28.1), university was instead modelled as a fixed effect to test for differences between schools.

First, we fitted a series of bivariable models containing the main effect of a factor plus time. Secondly, we included the interaction between the factor and time. Thirdly, the bivariable models were adjusted for the main effect of 1) age, 2) gender, 3) household composition, 4) prior nurse assistant, 5) prior work experience and 5) university, to discern the effect on the crude models (data not shown). Finally, to test that change in depressive symptoms over time remained after adding the effect of all investigated factors, we fitted multivariable models: a) a model of the main effects of all factors plus time, b) a series of models containing the main effects of all factors plus time, and the interaction of a specific factor and time and c) a model containing the main effect of all factors plus time, and the interactions between all factors and time. As we found an interaction between university and time, we tried to identify the source by recategorising the university variable into groups of large or small class size (> or < 70 students), type of school (university or university college) and university setting (large city or small/rural city where "large" refers to the three largest cities in Sweden). In addition we stratified for years in education (year 1, 2 and 3) and years from graduation (years 3, 4 and 5) to separate time in education from transition and establishment in the profession.

Due to positive skewness in the response variable, all analyses were rerun after log transformation of depressive symptoms, but as results were similar they are presented on the original scale. We chose not to impose any constraints on the covariance structure for the repeated measures at the individual level and used an unstructured matrix [29]. For university modelled as a random factor we used a variance components matrix, although it was not included in the final models presented here.

## Results

### The cohort at baseline

The age range at baseline was 20-52 (mean 28.42, median 26) with a similar gender distribution across all age groups. More than half of the students were married or cohabited with a partner and 40% were parents. Many had prior experience of work and higher education or were trained as nurse assistants before entering nursing education (see Table 1).

**Table 1:** Description of the cohort year 1 and 5

Time invariant factors	Year 1	Year 5	Retention*
	*N and (%)*	*N and (%)*	*(%) of cohort*

**Gender**			
Male	184 (10.82)	136 (10.53)	(73.91)
Female	1513 (89.18)	1156 (89.47)	(76.40)

**Age at baseline**			
20-24	711 (41.94)	519 (40.17)	(73.00)
25-29	351 (20.71)	263 (20.36)	(74.93)
30-34	263 (15.47)	211 (16.33)	(80.23)
35+	372 (21.88)	299 (23.14)	(80.38)

**Prior work experience before nursing education**			
Both within and outside health care	433 (25.47)	327 (25.31)	(75.52)
Health care only	582 (34.24)	458 (35.45)	(78.69)
Outside of health care only	482 (28.35)	354 (27.40)	(73.44)
No prior work experience	196 (11.53)	147 (11.38)	(75.00)
*Missing*	*4 (<0.01)*	*6 (<0.01)*	

**Prior training as nurse assistant**			
Yes	766 (45.06)	582 (45.05)	(75.98)
No	923 (54.29)	701 (54.26)	(75.95)
*Missing*	*8 (<0.01)*	*9 (<0.01)*	

**Prior experience of higher education**			
No prior experience	1260 (74.12)	954 (73.84)	(75.71)
A year or less	301 (17.71)	230 (17.80)	(76.41)
More than a year	124 (7.29)	96 (7.43)	(77.42)
*Missing*	*12 (<0.01)*	*12 (<0.01)*	

**University setting**			
Major city	373 (21.98)	292 (22.60)	(78.28)
Smaller city or rural area	1324 (78.02)	1000 (77.40)	(75.36)

**Time variant factor**			
	*N and (%)*	*N and %*	

**Household composition**			
Live alone	424 (24.94)	215 (16.64)	
Live with parents	96 (5.65)	9 (0.70)	
Live with partner	457 (26.88)	356 (27.55)	
Single with children	98 (5.76)	74 (5.73)	
Live with partner and children	563 (33.12)	635 (49.15)	
*Missing*	*59 (3.48)*	*3 (<0.01)*	

*Proportion completers in the group still in study year 5, compared with the defined cohort of 1700

### Drop out over time

The response rate in year five was 76% compared with baseline (see Figure 1. and Table 1.). Dropouts were younger (27.55 versus 28.69 years) and more often men, than completers, but this did not greatly affect the age or gender distribution. Dropouts before year five had a higher mean level of depressive symptoms at baseline compared with completers (8.25 versus 7.56, p = 0.02).

### Completion of education

**Figure 2:**
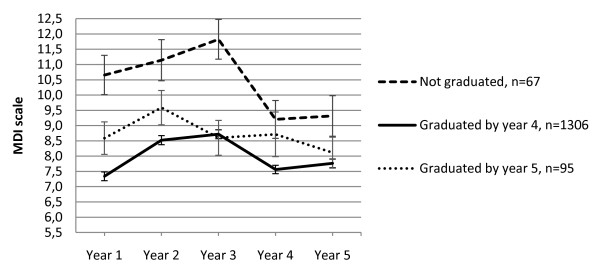
Graph of predicted means of depressive symptoms stratified for graduation and time of graduation*. *Estimates and error bars (of +/- 1 standard error) from a bivariable model of depressive symptoms over time containing the main effect for graduation year (year 4, 5 or no graduation within the study frame) plus time and the interaction between graduation year and time (n = 1468, the number of participants with information on graduation).

Respondents who graduated according to the expected time plan reported fewer depressive symptoms both at baseline and over time compared with late graduates and the few non-graduates. In addition, the groups showed different trajectories of depressive symptoms over time depending on graduation status (see Figure 2.). In year five 96.1% of 1292 remaining respondents had completed their education.

### Response profiles over time

**Figure 3:**
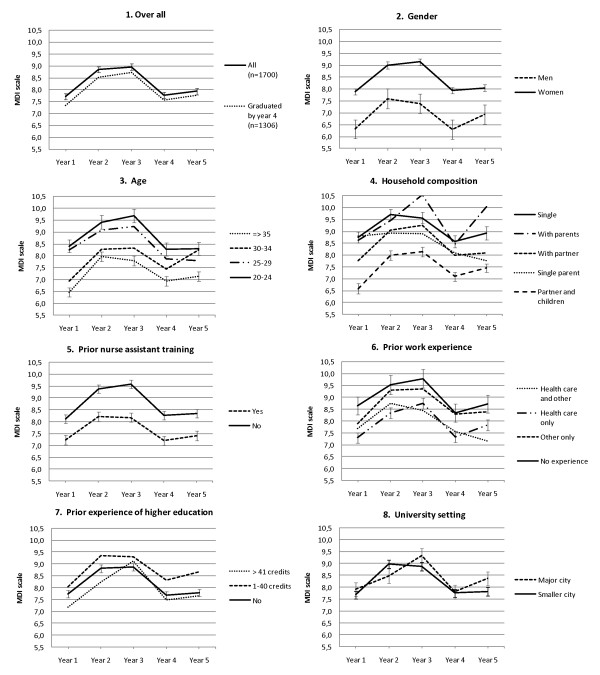
Graphs of predicted means of depressive symptoms over education (year 1-3) and establishment in the profession (year 4-5)*. *Graph 1: Estimates and error bars (of +/- 1 standard error) from two models of change in depressive symptoms over time in a) all 1700 cohort members and b) the subset of 1306 cohort members who graduated according to the expected time schedule, before year 4. **Graphs 2-8**: Estimates and error bars (of +/- 1 standard error) from bivariable models of depressive symptoms containing the main effect of a factor plus time, and the interaction between that factor and time (Bivariable models in Table 2.).

Graphs of depressive symptoms over time showed an increase from the first to the second and third year in education. After graduation and a year in the profession the symptoms appeared to decrease to a level similar to baseline. This pattern was similar across subgroups of all included factors, but the level of symptoms differed (see Figure 3.).

### Bivariable models

The change in mean level of depressive symptoms over time was significant (7.73, SD 5.29 year 1; 8.85, SD 5.39 year 2; 8.96, SD 5.18 year 3; 7.77, SD 4.97 year 4 and 7.94, SD 4.92 year 5; p = < 0.01). Bivariable models confirmed a difference in level of symptoms for subgroups of age, gender, household composition, prior nurse assistant training and prior work experience but also that there was no significant difference in change over time except for subgroups of age (see Table 2.). After adjustment for household composition the difference in change over time for subgroups of age was no longer significant (F 1.502, p = 0.136). There was no significant difference in level of symptoms between the 26 universities, but there was for change over time. Analyses stratified for year one to three versus year three to five showed this occurred during education. Recategorising into class size, type of school, and university setting, indicated a dissimilar development between year two and three for universities located in major cities compared with smaller cities (see Figure 3, Graph 8.).

**Table 2:** Significance tests for fixed effects from linear mixed effects models in all subjects (N = 1700)

		Bivariable models*					
		*Main effect only*		*Main effect and interaction*			
**Factor**	**Df**	**F**	***p***	**F**	***p***		

Year	4	1040.666	*< 0.001*				
Age	3	12.790	*< 0.001*	11.999	*< 0.001*		
Age*Year	12			2.057	*0.017*		
Gender	1	20.086	*< 0.001*	19.583	*< 0.001*		
Gender*Year	4			0.918	*0.453*		
Household composition	4	17.996	*< 0.001*	18.252	*< 0.001*		
Household composition*Year	16			0.799	*0.688*		
Prior nurse assistant training	1	20.586	*< 0.001*	20.836	*< 0.001*		
Prior nurse assistant training*Year	4			0.576	*0.680*		
Prior higher education	2	0.876	*0.417*	0.808	*0.446*		
Prior higher education*Year	8			0.602	*0.777*		
Prior work experience	3	4.438	*0.004*	4.375	*0.005*		
Prior work experience*Year	12			1.258	*0.238*		
University setting	1	3.495	*0.062*	2.775	*0.096*		
University setting*year	4			3.915	*0.004*		

		Multivariable models**				Multivariable model***	
		*Main effects only (a)*		*Main effects and interaction (b)*		*Main effects and interactions (c)*	

**Factor**	**Df**	**F**	***p***	**F**	***p***	**F**	***p***

Year	4	36.916	*< 0.001*			6.037	*< 0.001*
Age	3	2.526	*0.056*	2.442	*0.063*	2.191	*0.087*
Age*Year	12			1.337	*0.191*	1.660	*0.070*
Gender	1	26.765	*< 0.001*	26.312	*< 0.001*	26.002	*< 0.001*
Gender*Year	4			0.585	*0.674*	0.592	*0.669*
Household composition	4	14.201	*< 0.001*	14.281	*< 0.001*	12.433	*< 0.001*
Household composition*Year	16			1.087	*0.361*	1.107	*0.342*
Prior nurse assistant training	1	4.639	*0.031*	5.014	*0.025*	5.332	*0.021*
Prior nurse assistant training*Year	4			1.119	*0.346*	1.223	*0.299*
Prior higher education	2	0.585	*0.557*	0.634	*0.530*	0.596	*0.551*
Prior higher education*Year	8			0.719	*0.675*	0.653	*0.733*
Prior work experience	3	0.579	*0.629*	0.586	*0.624*	0.559	*0.642*
Prior work experience*Year	12			1.455	*0.135*	1.752	*0.051*
University setting	1	0.963	*0.326*	0.505	*0.477*	0.468	*0.494*
University setting*year	4			3.917	*0.004*	3.954	*0.003*

* **Main effect only**: Models containing the main effect of a factor plus time. **Main effect and interaction**: Models containing the main effect of a factor plus time and the interaction between the factor and time.

** **Main effects only (a)**: Model containing the main effects of all included factors plus time. **Main effects and interaction (b)**: Models containing the main effects of all included factors plus time and the interaction between one factor and time.

*** Model containing the main effects of all factors plus time and the interactions between all factors and time (c).

### Multivariable models

After adding the main effects for all factors in the same model (multivariable model a, see Table 2.), the difference in level of symptoms for different age groups and prior work experience were no longer significant. When the interaction between an individual factor and time was added (multivariable models b) there were no detectable differences in change over time across groups except for university setting (F 3.917, p = 0.004). Adding both main effects and interactions between factors and time for all factors in the same model (multivariable model c) did not change the results from multivariable models a and b.

The correlation among the repeated measures of depressive symptoms over time on the individual level varied between 0.49-0.60 for adjacent years and was 0.44 for the longest time lag from year one to year five in multivariable model c.

## Discussion

We found an increase of depressive symptoms from the first to later years in higher education, and a decrease to a level similar to baseline after graduation when a majority of the former students had worked professionally for at least a year. Age, gender, household composition and prior training as nurse assistant affected the reported levels of depressive symptoms, but change over time was similar in all groups. There was a difference in development over time between universities, but overall the results imply an effect of time in higher education and professional establishment on depressive symptoms, regardless of age, gender and prior experience. The correlation among the repeated measures over time suggests that individual differences in levels of depressive symptoms persist.

### Change of depressive symptoms over time

The literature on psychological distress by time in education we encountered come from a limited number of education programs, but despite differences in length and choice of profession, students in medical [7,30], dental [6,8], law [9] and nursing education [15-17] often show elevated levels of psychological distress or depression in the final year compared with initial levels. Some studies measured distress only at the beginning and end of education, but those that followed all years appear to agree in that they show a greater increase from the first to second year after which levels remain more or less stable until graduation [7-9,16]. In our material we found a slight difference in symptom development over time in education by university for schools located in major versus smaller cities but both curves indicate heightened distress over education and a decrease after graduation.

After education, longitudinal studies in recently graduated medical doctors point to high initial levels of distress that decline over the first years in the profession [31,32] and qualitative studies in nurses consistently show new graduates subject to high initial stress as they encounter the profession, but also a gain in professional confidence over the first year at work [18,33,34]. Our results indicate approximately stable levels of symptoms the first two years in the profession, but as most respondents worked for at least a year before they answered the questionnaire we may have missed an initial peak of distress.

We found one study that compared distress development in a cohort of students with a cohort of newly qualified professionals from the same college, incidentally in similarity to our data, performed in nursing students. Contrary to us, the study reported higher psychological distress (measured by the GHQ-12) over the first four professional years as nurses than in nursing students at any point in education [35]. In difference, we were able to follow a single cohort of students from multiple nursing colleges over the complete period.

Overall, the findings seem to agree with prior research in students and new graduates and in addition, due to the extended follow-up of the former students after education, we were able to join results from education and professional establishment. We then saw a transitional pattern of elevated distress in the latter part of education that decreased once the graduate had had time to accommodate to the occupation. However, beneficial development during professional establishment may also depend on other factors such as employment prospects and job security after graduation [36]. The new graduates in the present study encountered a labour market of less than one percent unemployment among registered nurses [37].

### Difference in levels of depressive symptoms between groups

The differences found in symptom levels reflect results on depression from population based American and European surveys: a higher prevalence among young adults compared with middle aged adults, in women compared with men, and among singles compared with married/cohabiters [38-40]. In addition, prior experience from health care before nursing education; training as nurse assistant at high school level and/or work within health care were associated with lower levels of depressive symptoms. Perhaps the choice of entering nursing education differed between groups: students with prior experience of health care may have been more prepared for training and work within health care.

Thus, we found similar associations between the investigated demographic factors and depressive symptoms in students as has been found for depression in the general population. That, despite differences in level of symptoms, these factors had little impact on change in depressive symptoms over time, supports the hypothesis of an independent effect of time in higher education and professional establishment on distress.

### Correlation over time

The correlation between the repeated measures suggests that individual differences in symptom level between responders persist. This is in line with longitudinal data from both general student samples, where students who reported distress at baseline had an increased risk for depression two years later [41] and medical students, where high distress in first year students predicted psychological morbidity in late education [42].

### Clinical significance

By investigating symptom degree rather than depressive disorder, we could follow change over time but not the proportion of students afflicted by clinical levels of depression. To assess the clinical relevance of the findings we calculated a DSM-IV based measure of presence versus absence of depression from the MDI-scale. This showed an estimated proportion of 10.1% depressed the first year of education and a pattern of change over time similar to that found for levels of symptoms (12.2% depressed year 2, 11.3% year 3 and 7.7% year 4 and 5). Despite this, the standard deviations of 4.92-5.39 over time display that even if we found a consistent and significant change in mean levels of symptoms, it is modest compared with variation on the individual level each point in time. We think the results we present have implications on an aggregate level and that they do indicate an effect of education and professional establishment on depressive symptoms, but that for individuals there are other and more important factors that affect depression.

### Strengths and limitations

The nation-wide design and the demographic make-up of the student group in this study enabled analysis of differences in distress development between nursing programmes and control for differences due to age, living conditions and prior experience. The analysis was restricted to a single profession which limits inference to other professions, but studies performed in other student groups in professional training seem to indicate similar development over time. As for students who do not pursue organised programs they may have different goals with less defined outcomes in terms of a future career, which perhaps affect distress both over education and after graduation differently.

The response rate of 73% at baseline was comparable or higher than other studies on mental health in students we know of, and attrition from the defined cohort over time was modest although there were some differences between groups over time. As for change over education and professional establishment the respondents were already in their second term at first measurement and a year into work life at the last, consequently we may have missed initial distress both in beginner students and new graduates. We located a difference in distress development for university setting; but there are other plausible causes we were unable investigate. This said, our impression after inspecting descriptive graphs of development for individual schools, is that the pattern of increase between year one and two and decrease after examination and a year in the profession holds true over a majority of schools (see additional files 1 and 2: "Depressive symptoms by year and university" and "Depressive symptoms by year and university stratified for class size, type of school and university setting").

In LANE, 95.3% of the remaining respondents had graduated by year five, but national registers show that only 83% of those who initiated nursing education in 2001/2002 graduated within five years [43]. This means respondents in year five were a selection of students who completed their studies and we cannot assume non-response either at baseline or over time to be missing completely at random (MCAR). Completers may also be a selection of participants less prone to distress and depression to begin with. The results may be subject to a "healthy worker" effect, and the estimates of depressive symptoms biased downwards by time. We stratified the analyses for graduation within four years and compared results with a complete cases analysis and found a slight difference in level of symptoms, but as the developmental curve over time remained the assumption of missing at random (MAR) underlying the analyses is supported.

## Conclusions

The fact that change in depressive symptoms over time was similar across groups indicates an independent effect of time in higher education and professional establishment on psychological distress. We think heightened distress over education is a transitional phenomenon and that in the majority of former students it abates once they have had time to establish themselves in the profession.

These findings have implications for how to interpret psychological distress in student samples. Students in higher education are under constant evaluation and after graduation, as they enter occupational life, they have to prove themselves capable in their chosen profession. Heightened distress in late education may be anticipatory worry as well as a response to the immediate educational environment. This can be true of all students in higher education, but is perhaps easier to identify in professional education programs where a successfully accomplished degree defines the skills a graduate is expected to master.

Despite the transitional nature; within education, the differences observed for depressive symptoms in association to demographic factors can help identify student groups more vulnerable to distress. And as individual differences in distress seem to persist, perhaps students highly distressed in the beginning of education can be helped by awareness among educators and counsellors of the elevated levels of distress in late education.

## Abbreviations

OECD: Organisation for Economic Co-operation and Development; DSM-IV: Diagnostic and Statistical Manual of Mental Disorders, Fourth Edition; ICD-10: International Classification of Diseases and Related Health Problems, 10th Revision.

## Competing interests

The authors declare that they have no competing interests.

## Authors' contributions

AC participated in the questionnaire development, co-ordinated three of the data collections, designed the study, performed the statistical analyses and drafted the manuscript. BR was the guarantor of the study, supervised the first author and helped draft the manuscript. PD supervised, participated in and helped interpret results from the statistical analyses. MV supervised the first author and helped draft the manuscript. All authors read and approved the final manuscript.

## Pre-publication history

The pre-publication history for this paper can be accessed here:

http://www.biomedcentral.com/1471-2458/10/343/prepub

## Supplementary Material

Additional file 1 **Depressive symptoms by year and university**. Descriptive graph of the observed levels of depressive symptoms by year and university Click here for file

Additional file 2 **Depressive symptoms by year and university stratified for class size, type of school and university setting**. Descriptive graphs of the observed levels of depressive symptoms by year and university stratified for class size, type of school and university setting Click here for file

## References

[B1] Dyrbye LN Thomas MR Shanafelt TD Systematic review of depression, anxiety, and other indicators of psychological distress among U.S. and Canadian medical students Acad Med 2006 81 354 373 10.1097/00001888-200604000-00009 16565188

[B2] Webb E Ashton CH Kelly P Kamali F Alcohol and drug use in UK university students Lancet 1996 348 922 925 10.1016/S0140-6736(96)03410-1 8843811

[B3] Weitzman ER Poor mental health, depression, and associations with alcohol consumption, harm, and abuse in a national sample of young adults in college J Nerv Ment Dis 2004 192 269 277 10.1097/01.nmd.0000120885.17362.94 15060400

[B4] Cotton SJ Dollard MF de Jonge J Stress and student job design: Satisfaction, well-being, and performance in university students Int J Stress Manag 2002 9 147 162 10.1023/A:1015515714410

[B5] Stewart-Brown S Evans J Patterson J Petersen S Doll H Balding J Regis D The health of students in institutes of higher education: an important and neglected public health problem? J Public Health Med 2000 22 492 499 10.1093/pubmed/22.4.492 11192277

[B6] Gorter R Freeman R Hammen S Murtomaa H Blinkhorn A Humphris G Psychological stress and health in undergraduate dental students: fifth year outcomes compared with first year baseline results from five European dental schools Eur J Dent Educ 2008 12 61 68 10.1111/j.1600-0579.2008.00468.x 18412732

[B7] Rosal MC Ockene IS Ockene JK Barrett SV Ma Y Hebert JR A longitudinal study of students' depression at one medical school Acad Med 1997 72 542 546 10.1097/00001888-199706000-00022 9200590

[B8] Stewart DW de Vries J Singer DL Degen GG Wener P Canadian dental students' perceptions of their learning environment and psychological functioning over time J Dent Educ 2006 70 972 981 16954419

[B9] Reifman A McIntosh DN Ellsworth PC Depression and affect among law students during law school: A longitudinal study J Emot Abuse 2001 2 93 106 10.1300/J135v02n01_07

[B10] Swedish National Agency for Higher Education Universitet & Högskolor. Högskoleverkets årsrapport 2009. Official Statistics of Sweden: Högskoleverkets rapportserie 2009:12 R. (in Swedish) http://www.hsv.se/download/18.1dbd1f9a120d72e05717ffe2356/0912R.pdf (accessed on 27 November 2009)

[B11] Eisenberg D Gollust SE Golberstein E Hefner JL Prevalence and correlates of depression, anxiety, and suicidality among university students Am J Orthopsychiatry 2007 77 534 542 10.1037/0002-9432.77.4.534 18194033

[B12] Hankin BL Abramson LY Moffitt TE Silva PA McGee R Angell KE Development of depression from preadolescence to young adulthood: emerging gender differences in a 10-year longitudinal study J Abnorm Psychol 1998 107 128 140 10.1037/0021-843X.107.1.128 9505045

[B13] Blanco C Okuda M Wright C Hasin DS Grant BF Liu SM Olfson M Mental health of college students and their non-college-attending peers: Results from the National Epidemiologic Study on Alcohol and Related Conditions Arch Gen Psychiatry 2008 65 1429 1437 10.1001/archpsyc.65.12.1429 19047530 PMC2734947

[B14] Arnett JJ Emerging adulthood. A theory of development from the late teens through the twenties Am Psychol 2000 55 469 80 10.1037/0003-066X.55.5.469 10842426

[B15] Deary IJ Watson R Hogston R A longitudinal cohort study of burnout and attrition in nursing students J Adv Nurs 2003 43 71 81 10.1046/j.1365-2648.2003.02674.x 12801398

[B16] Lo R A longitudinal study of perceived level of stress, coping and self-esteem of undergraduate nursing students: an Australian case study J Adv Nurs 2002 39 119 126 10.1046/j.1365-2648.2000.02251.x 12100655

[B17] Lindop E A comparative study of stress between pre- and post-Project 2000 students J Adv Nurs 1999 29 967 973 10.1046/j.1365-2648.1999.00974.x 10215990

[B18] Gerrish K Still fumbling along? A comparative study of the newly qualified nurse's perception of the transition from student to qualified nurse J Adv Nurs 2000 32 473 480 10.1046/j.1365-2648.2000.01498.x 10964197

[B19] Jasper M The first year as a staff nurse: the experiences of a first cohort of Project 2000 nurses in a demonstration district J Adv Nurs 1996 24 779 790 10.1046/j.1365-2648.1996.25517.x 8894896

[B20] Oermann MH Garvin MF Stresses and challenges for new graduates in hospitals Nurse Educ Today 2002 22 225 230 10.1054/nedt.2001.0695 12027604

[B21] O'Shea M Kelly B The lived experiences of newly qualified nurses on clinical placement during the first six months following registration in the Republic of Ireland J Clin Nurs 2007 16 1534 42 10.1111/j.1365-2702.2006.01794.x 17655542

[B22] Ahmadi J Toobaee S Alishahi M Depression in nursing students J Clin Nurs 2004 13 124 10.1046/j.1365-2702.2003.00894.x 14687305

[B23] Haack MR Stress and impairment among nursing students Res Nurs Health 1988 11 125 134 10.1002/nur.4770110208 3363176

[B24] Ross R Zeller R Srisaeng P Yimmey S Somchid S Sawatphanit W Depression, stress, emotional support, and self-esteem among baccalaureate nursing students in Thailand Int J Nurs Educ Scholarsh 2005 2 1 15 10.2202/1548-923x.1165 16646920

[B25] Williams RA Hagerty BM Murphy-Weinberg V Wan J Symptoms of depression among female nursing students Arch Psychiatr Nurs 1995 9 269 278 10.1016/S0883-9417(95)80046-8 7487168

[B26] Gustavsson P Svärdson Å Lagerström M Bruce M Christensson A Schüldt-Håård U Omne-Pontén M Longitudinell undersökning av sjuksköterskors tillvaro (in Swedish). Karolinska Institutet, Stockholm http://ki.se/content/1/c6/03/22/59/serieB07no1.pdf (accessed on 27 November 2009)

[B27] Bech P Rasmussen NA Olsen LR Noerholm V Abildgaard W The sensitivity and specificity of the Major Depression Inventory, using the Present State Examination as the index of diagnostic validity J Affect Disord 2001 66 159 164 10.1016/S0165-0327(00)00309-8 11578668

[B28] Olsen LR Jensen DV Noerholm V Martiny K Bech P The internal and external validity of the Major Depression Inventory in measuring severity of depressive states Psychol Med 2003 33 351 6 10.1017/S0033291702006724 12622314

[B29] Fitzmaurice GM Laird NM Ware JH Fitzmaurice GM, Laird NM, Ware JH Modelling the covariance Applied Longitudinal Analysis 2004 Hoboken, New Jersey: John Wiley & Sons, Inc 163 185

[B30] Niemi PM Vainiomaki PT Medical students' distress--quality, continuity and gender differences during a six-year medical programme Med Teach 2006 28 136 141 10.1080/01421590600607088 16707294

[B31] Tyssen R Vaglum P Mental health problems among young doctors: an updated review of prospective studies Harv Rev Psychiatry 2002 10 154 165 10.1080/10673220216218 12023930

[B32] Virtanen P Rantalaiho L Koivisto AM Employment status passages and psychosocial well-being J Occup Health Psychol 2003 8 123 130 10.1037/1076-8998.8.2.123 12703878

[B33] Kelly B Preserving moral integrity: a follow-up study with new graduate nurses J Adv Nurs 1998 28 1134 1145 10.1046/j.1365-2648.1998.00810.x 9840887

[B34] Wangensteen S Johansson IS Nordstrom G The first year as a graduate nurse--an experience of growth and development J Clin Nurs 2008 17 1877 1885 10.1111/j.1365-2702.2007.02229.x 18578762

[B35] Watson R Gardiner E Hogston R Gibson H Stimpson A Wrate R Deary I A longitudinal study of stress and psychological distress in nurses and nursing students J Clin Nurs 2009 18 270 278 10.1111/j.1365-2702.2008.02555.x 19120753

[B36] Virtanen P Koivisto A Wellbeing of professionals at entry into the labour market: A follow up survey of medicine and architecture students J Epidemiol Community Health 2001 55 831 835 10.1136/jech.55.11.831 11604440 PMC1763309

[B37] The National Board of Health and Wellfare Statistics on Health Care Personnel - Official Statistics on the Number of Licensed Practitioners (2006) and their Labour Market Situation (2005). Official Statistics of Sweden: Statistics - Health Care 2007:2. (in Swedish) http://www.socialstyrelsen.se/Lists/Artikelkatalog/Attachments/9350/2007-46-3_2007463.pdf (accessed on 27 November 2009)

[B38] Alonso J Angermeyer MC Bernert S Bruffaerts R Brugha TS Bryson H de Girolamo G Graaf R Demyttenaere K Gasquet I Haro JM Katz SJ Kessler RC Kovess V Lepine JP Ormel J Polidori G Russo LJ Vilagut G Almansa J Arbabzadeh-Bouchez S Autonell J Bernal M Buist-Bouwman MA Codony M Domingo-Salvany A Ferrer M Joo SS Martinez-Alonso M Matschinger H Mazzi F Morgan Z Morosini P Palacin C Romera B Taub N Vollebergh WAM EsemeD/Mhedea Investigators European Study of the Epidemiology of Mental Disorders Project Prevalence of mental disorders in Europe: results from the European Study of the Epidemiology of Mental Disorders (ESEMeD) project Acta Psychiatr Scand Suppl 2004 420 21 27 15128384 10.1111/j.1600-0047.2004.00327.x

[B39] Kessler RC Berglund P Demler O Jin R Koretz D Merikangas KR Rush AJ Walters EE Wang PS The epidemiology of major depressive disorder: results from the National Comorbidity Survey Replication (NCS-R) JAMA 2003 289 3095 3105 10.1001/jama.289.23.3095 12813115

[B40] Patten SB Wang JL Williams JV Currie S Beck CA Maxwell CJ el-Guebaly N Descriptive Epidemiology of Major Depression in Canada Can J Psychiatry 2006 51 84 90 16989107 10.1177/070674370605100204

[B41] Zivin K Eisenberg D Gollust SE Golberstein E Persistence of mental health problems and needs in a college student population J Affect Disord 2009 117 180 185 10.1016/j.jad.2009.01.001 19178949

[B42] Guthrie E Black D Bagalkote H Shaw C Campbell M Creed F Psychological stress and burnout in medical students: a five-year prospective longitudinal study J R Soc Med 1998 91 237 243 9764076 10.1177/014107689809100502 PMC1296698

[B43] Statistics Sweden. Higher Education Throughput and result in undergraduate education up to 2003/04 inclusive. Official Statistics of Sweden: Utbildning och forskning UF20SM0502 (in Swedish) http://www.scb.se/statistik/UF/UF0205/2005A05/UF0205_2005A05_SM_UF20SM0502.pdf (accessed on 27 November 2009)

