# Supplementary material for: Change in depressive symptoms over higher education and professional establishment - a longitudinal investigation in a national cohort of Swedish nursing students
Source: BMC Public Health. 2010 Jun 15;10:343. doi: 10.1186/1471-2458-10-343 (PMC2905329; doi:10.1186/1471-2458-10-343)
Supplement: Additional file 1 — Depressive symptoms by year and university. Descriptive graph of the observed levels of depressive symptoms by year and university [file 1471-2458-10-343-S1.PDF]

Additional file 1. Depressive symptoms by year and university*

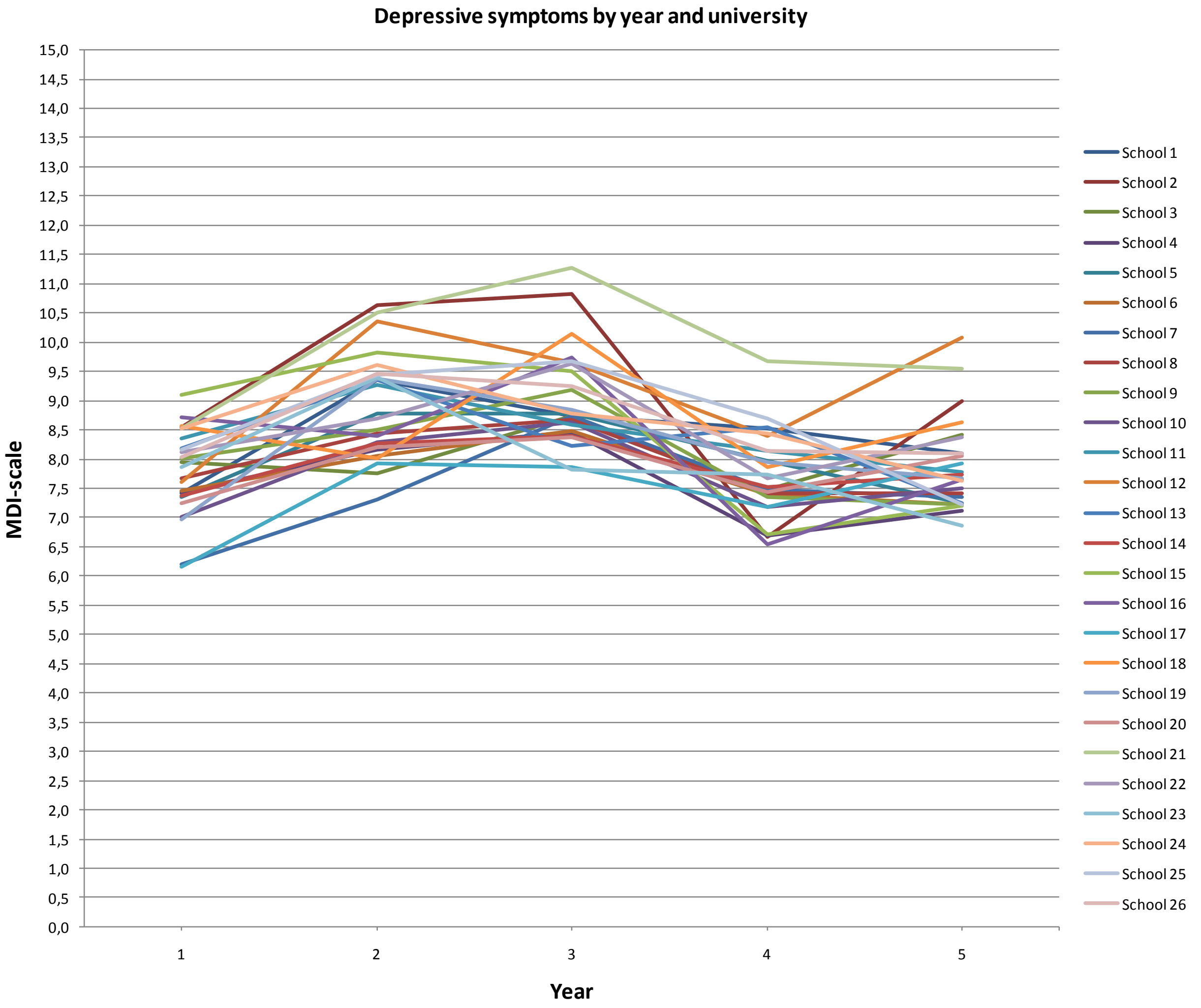

**Please note this is a descriptive graph of observed levels of symptoms and that, apart from differences in participation rates at baseline and attrition over time, the number of respondents per school vary from 13-150 year one and 9-120 year five.*
