# Supplementary material for: Change in depressive symptoms over higher education and professional establishment - a longitudinal investigation in a national cohort of Swedish nursing students
Source: BMC Public Health. 2010 Jun 15;10:343. doi: 10.1186/1471-2458-10-343 (PMC2905329; doi:10.1186/1471-2458-10-343)
Supplement: Additional file 2 — Depressive symptoms by year and university stratified for class size, type of school and university setting. Descriptive graphs of the observed levels of depressive symptoms by year and university stratified for class size, type of school and university setting [file 1471-2458-10-343-S2.PDF]

Additional file 2. Depressive symptoms by year and university stratified for class size, type of school and university setting*

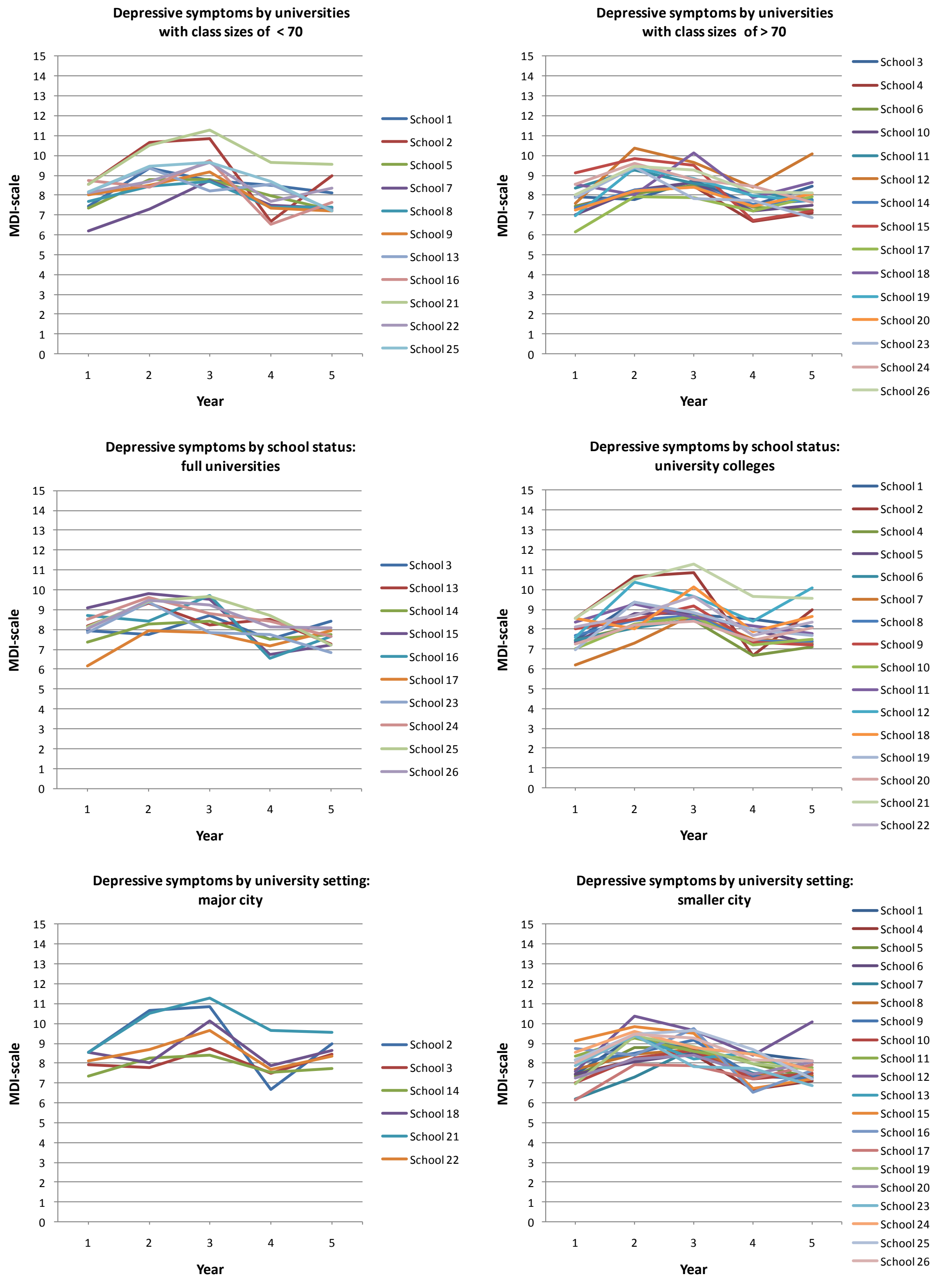

**Please note these are descriptive graphs of observed levels of symptoms and that, apart from differences in participation rates at baseline and attrition over time, the number of respondents per school vary from 13-150 year one and 9-120 year five.*
